# An Update on the Special Issue “Parent-Child Interactions: Paths of Intergenerational Transmission of Psychopathological Risk”

**DOI:** 10.3390/ijerph21030328

**Published:** 2024-03-12

**Authors:** Luca Cerniglia

**Affiliations:** Faculty of Psychology, International Telematic University Uninettuno, 00186 Rome, Italy; luca.cerniglia@uninettunouniversity.net; Tel.: +39-06697621

In September 2020, this Journal published a Special Issue (SI) entitled “Parent-Child Interactions: Paths of Intergenerational Transmission of Psychopathological Risk” that included fourteen interesting articles (see here for all of the published manuscripts’ references: https://www.mdpi.com/journal/ijerph/special_issues/Parent_Child_Interactions (accessed on 5 February 2024). All of the manuscripts were consistent with the bio-psycho-social model, offering a multidimensional perspective that integrated biological predispositions, psychological states, and social environments in understanding the nuanced mechanisms underlying the intergenerational transmission of psychopathological risks [1,2,3,4,5].

At the heart of this discussion was the acknowledgment of parental psychopathology as a significant predictor of maladaptive developmental outcomes in their offspring. Drawing upon a robust body of evidence, including animal studies, the papers underscored the critical role of the parental care environment in shaping long-term effects on neural systems responsible for stress regulation, emotional function, and neuroplasticity in children. These findings emphasized the biological underpinnings of inherited psychopathological risks while also highlighting the potential for environmental and social factors to mitigate or exacerbate these inherited vulnerabilities.

The notion of caregiving quality emerged as a crucial factor in this field. The authors posited that epigenetic mechanisms, influenced by the quality of parental caregiving, could mediate the relationship between parental psychopathology and offspring development. This assertion opened new avenues for understanding how modifications in gene expression, driven by environmental factors, could have lasting effects on child development, independent of the child’s genetic disposition.

Furthermore, the discussion extended to the importance of sensitive and attuned interactions between parents and children. Such interactions were fundamental to fostering emotional and behavioral regulation in children, thereby serving as a protective factor against the development of maladaptive symptoms. This perspective was supported by research emphasizing the bidirectional nature of parent–child exchanges, highlighting the role of temperament and the child’s innate predispositions in influencing these interactions. The Special Issue also addressed the challenges posed by the COVID-19 pandemic [2,3,4,5,6,7,8,9], particularly its impact on parent–child dynamics. This context added a layer of complexity to the already intricate relationship between parental psychopathology and child development, prompting a reevaluation of interaction quality assessment methodologies. The SI called for innovative methodological approaches to capture the nuances of these interactions under the unique stresses imposed by the pandemic.

In response to these challenges, the Special Issue presented a collection of research papers and reviews that explored various dimensions of parent–child interactions [10,11,12,13,14,15,16]. Among the highlighted studies was an investigation into the effects of Focal Play Therapy with Children and Parents, which revealed significant improvements in parent–child interactions post-intervention. This finding underscored the potential of targeted interventions to enhance the quality of caregiving and, by extension, child developmental outcomes.

Another noteworthy contribution explored the bidirectional links between depressive symptoms in caregivers and adolescents, employing a transactional framework and complex statistical models. This study offered critical insights into the interplay between caregiver mental health and adolescent well-being, further emphasizing the interconnectedness of family dynamics in shaping psychological outcomes.

In synthesizing these contributions, the editorial not only shed light on the multifaceted nature of parent–child interactions but also underscored the critical need for a holistic approach to addressing psychopathological risk transmission. The call for methodological innovation reflected a broader imperative for research that could adapt to evolving social and environmental contexts, including the unprecedented challenges of the COVID-19 pandemic. By integrating findings from diverse studies, the Special Issue contributed significantly to the body of knowledge on parent–child interactions, offering valuable perspectives for both clinical practice and future research. The emphasis on innovative assessment methodologies, along with the exploration of interventions and their impact on family dynamics, provided a roadmap for advancing the field in ways that were responsive to both the inherent complexities of parent–child relationships and the external challenges posed by societal changes.

Although very interesting, all the articles recognized limitations in their own articulation. For example, Cimino et al.’s, Chirico et al.’s, and Khoury et al.’s manuscripts advised on the importance of confirming their results in larger samples. More recent papers have addressed this expectation by recruiting larger samples and demonstrated that genetic factors, in conjunction with parental behavior, play a significant role in the development of executive functions in children, which can be related to emotional–behavioral regulation and temperament. Moreover, further studies found that Focal Play Therapy contributes to the understanding of the dynamics between parent–child interactions, the impact of parental stress, and the effectiveness of interventions aimed at improving these relationships. In addition, Niu et al. [17] confirmed Khoury’s results concerning the importance of interventions aimed at improving parent–child relationships and addressing the consequences of maltreatment across generations.

A group of other articles in the Special Issue (authored by Trentini et al.; Coppola et al.; Yu et al.; Hou et al.; Agostini et al.; Cerniglia et al.) acknowledged that their studies did not consider other key variables that should have been taken into consideration for more robust results. The literature has now filled this gap, at least partially, and in particular, Wendelboe et al. [18] have shown that higher levels of reflective function were associated with lower levels of parenting stress, providing further evidence for the assessment of paternal reflective skills. Moreover, Cui et al. [19] found that children’s traits, including narcissism, can influence parental behavior, as parents adjust their parenting styles in response to their children’s temperament and behaviors. McGill et al. [20] showed how financial stress impacts parenting behaviors and child well-being, particularly under the strains of economic hardship and global crises like the COVID-19 pandemic. Further, Rivers et al. [21] demonstrated the key role of maternal attachment as a predictor of depressive symptoms, highlighting the unique importance of maternal attachment in understanding internalizing symptoms among high-risk adolescents. Paterlini et al. [22], collaborating with the same authors of the paper presented in the SI, posited that the psychological well-being of parents who conceived after Assisted Reproductive Treatments (ARTs) is central and should be investigated.

Two studies suggested the usefulness of longitudinal studies regarding the implementation of a physical therapy stimulation program for children with disabilities and reading deficits in young children during the COVID-19 pandemic. Bailes et al. [23] demonstrated the effectiveness of physical therapy and stimulation programs for children with disabilities in a longitudinal study, and Shult et al. [24] showed that longer periods of school closures were associated with larger learning losses for a group of low-achieving students and schools with less socio-cultural capital.

Sacchi et al.’s and Speranza et al.’s manuscripts stimulated subsequent literature to confirm the validity of the measures used in studies focusing on parental emotional state and child behavior. Although not directly responding to Sacchi’s work, one notable paper has been published in recent years on these topics, partially filling the presented gap: Grumi et al. [25] published an interesting article on a video–feedback intervention. Quintiliano et al. [26] proposed a new study on parent–child relationships in infancy and early childhood.

Lastly, Wong et al. underlined the importance of publishing systematic reviews on the topic of intergenerational resource transfer. In this regard, Capolupo et al.’s [27] paper can be cited as a recently published relevant article.

At the publication of this paper, the articles included in the Special Issue have been cited 151 times as a whole, and the manuscripts have received more than sixty-thousand views, demonstrating strong interest from academics in the field of Developmental Psychopathology [28,29,30,31,32,33].
